# Feasibility study of using the PREDICT kidney tool for patients with localised renal cell carcinoma

**DOI:** 10.1002/bco2.70014

**Published:** 2025-03-30

**Authors:** Panayiotis Laouris, Chiara Re, Georgia Stimpson, Axel Bex, James Blackmur, Alexander Laird, Carley Batley, Grant D. Stewart, Hannah Harrison, Juliet A. Usher‐Smith

**Affiliations:** ^1^ Department of Public Health and Primary Care University of Cambridge Cambridge UK; ^2^ Department of Surgery University of Cambridge, Addenbrooke's Hospital Cambridge UK; ^3^ Royal Free London NHS Foundation Trust, UCL Division of Surgery and Interventional Science London UK; ^4^ Western General Hospital, NHS Lothian Edinburgh UK; ^5^ The Institute of Genetics and Cancer The University of Edinburgh Edinburgh UK; ^6^ Department of Oncology University of Cambridge, Addenbrooke's Hospital Cambridge UK

**Keywords:** feasibility trial, kidney cancer, recurrence risks, risk communication, risk estimates

## Abstract

**Background:**

Localised renal cell carcinoma (RCC) is usually treated surgically, with post‐operative imaging‐based surveillance to monitor for recurrence. However, surveillance practices vary widely, and patients often lack a clear understanding of their risk of recurrence and follow‐up care. The PREDICT Kidney tool has been developed to enhance risk communication by providing individualised recurrence and mortality risk estimates. The tool uses the Leibovich score augmented with English national data to provide a personalised risk assessment of cancer recurrence and death from other causes, presented in both numerical and visual formats.

**Study Design:**

A multicentre, prospective feasibility study of incorporating the PREDICT Kidney risk communication tool into the first follow‐up consultation for localised RCC patients post‐surgery.

**Endpoints:**

Patient uptake into the study, completeness of data collection, consultation duration, the acceptability of the tool to both patients and clinicians, clinician adherence to the study “best‐practice” guide, variability in tool usage across clinicians and sites and patient‐level clinical outcomes including subjective and objective comprehension of risk of recurrence and follow‐up, perceived risk of cancer recurrence, risk conviction, satisfaction with the information provided on risk of recurrence and follow‐up, and fear of cancer recurrence.

**Patients and Methods:**

We aim to recruit 60 patients from three hospitals in England and Scotland. Patients treated with surgery for primary localised clear‐cell RCC awaiting their first follow‐up appointment will be invited to take part. Participants will be allocated into two groups: standard care and standard care supplemented with the use of the PREDICT Kidney tool. Data will be collected through questionnaires, audio/video recordings of consultations and interviews with a subset of patients and clinicians. The study period is planned from September 2024 to July 2025. The findings will guide the design of a future randomised controlled trial to evaluate the tool's efficacy in clinical settings.

## 1 BACKGROUND

Renal cell cancer (RCC) is the 7th most common cancer in the UK^1^ and its management increasingly requires a multi‐specialist approach.^2^ Between 70% and 75% of patients diagnosed with RCC have localised disease at diagnosis and are most commonly treated surgically^3^; however, approximately 20% subsequently develop cancer recurrence.^4^ Surveillance, including interval imaging with computerised tomography (CT) scans, is an important component of post‐operative care for these patients. However, surveillance is associated with potential harm for patients, including anxiety and radiation exposure, as well as being resource intensive for the healthcare service.

Tailoring surveillance based on individual risk of recurrence is recommended by many current guidelines,^5^, ^6^, ^7^, ^8^, ^9^ with the recommended follow‐up schedules for patients differing with respect to modality, frequency and duration depending on risk classification. No guideline recommends one specific risk model or risk threshold, leaving the choice to clinicians.^10^ In the UK, one of the most widely used risk models, which is also recommended in the recently published Getting It Right First Time (GIRFT) guidelines,^11^ is the Leibovich score.^12^ This score estimates the risk of recurrence based on tumour characteristics (size, stage, nuclear grade, presence of necrosis and regional lymph node status). Based on their score, patients are stratified into low (score 0–2), intermediate (score 3–5) and high (score above 6) risk groups for cancer recurrence, which subsequently guides their surveillance. Although the estimated metastasis‐free survival rate for the three risk categories has been published, and the score has been validated in studies for recurrence‐free survival, cancer‐specific survival and overall survival,^13^ it does not give an absolute risk of recurrence or take into consideration other competing health risks. Surveillance is also a source of anxiety for many patients^14^ and understanding of decision‐making about follow‐up is poor. In a recent study, in which we held focus groups with patients with kidney cancer to understand their experience of follow‐up care,^15^ participants described marked variation in how the risk of recurrence was communicated and a lack of transparency around decision‐making for risk‐stratified follow‐up. Some were able to recall being given their Leibovich score, whilst others reported no conversations with their clinical team about their risk of recurrence. Fear of recurrence is common in patients with localised RCC, with this affecting quality of life and health outcomes.^16^ It is also known from studies in other cancer types that patient recall of risk is often poor, with many perceiving their risk to be higher than the actual estimate.^17^

To address these issues, our team has developed PREDICT Kidney, a new online tool that presents the absolute risk of recurrence based on the Leibovich score alongside the competing age‐ and sex‐matched absolute risk of death from other causes. The tool has been co‐designed by patients, clinicians and members of the public to be used in consultations to support clinicians when communicating the risk of recurrence, with the aim that patients feel more informed about their risk of recurrence and have a better understanding of the rationale behind their planned follow‐up.

## 2 STUDY DESIGN

This study is a multicentre feasibility study of incorporating the PREDICT Kidney tool into the first follow‐up consultation post‐surgery in patients with newly diagnosed localised renal cell carcinoma.

## 3 ENDPOINTS

The overall aims of this study are to (a) assess the feasibility and acceptability of incorporating the PREDICT Kidney online tool into the first post‐surgery follow‐up appointments for patients with localised renal cell carcinoma and (b) to evaluate participant recruitment, completeness of data collection and estimates of patient‐level outcomes to inform the design of a future trial. The study objectives and outcome measures are described in Table 1.

**TABLE 1:** Overview of feasibility and patient‐level objectives, and their corresponding outcomes and time‐points.

Objective	Outcome	Time point
To quantify uptake into the study	The proportion of eligible patients invited to participate who consent to take part	At completion of recruitment
To assess the completeness of data collection	The proportion of patients who consent to participate in trial who go on to complete the questionnaires	At the completion of recruitment
To measure the time needed to use the tool and any change in overall length of consultation	The delivery time of the PREDICT Kidney tool during consultation appointments (intervention group only) The length of consultation appointments	Within first follow‐up consultation
To assess the acceptability of integrating the tool into clinical care to patients and clinicians	Acceptability to patients and clinicians	At interview (2–6 weeks after first follow‐up consultation)
To assess adherence to the study “best‐practice” guide by clinicians and variability between clinicians and sites in use of the tool	Clinician adherence to “best‐practice” checklist	Within first follow‐up consultation
To evaluate patient‐level clinical outcomes	Satisfaction with the information provided on risk of recurrence and follow‐up	Immediately after first follow‐up consultation
	Subjective comprehension of risk of recurrence and follow‐up	Immediately after first follow‐up consultation
	Objective comprehension of risk of recurrence	Immediately after first follow‐up consultation and at 3‐months post consultation
	Perceived risk of cancer recurrence	3‐months post consultation
	Risk conviction	Immediately after first follow‐up consultation and at 3 months post consultation
	Fear of cancer recurrence	Immediately after first follow‐up consultation and at 3 months post consultation

## 4 ELIGIBLITY CRITERIA

Participants will be eligible for inclusion in the study if they have been treated with surgery (either partial or radical nephrectomy) for primary localised clear cell RCC and are awaiting their first post‐surgery follow‐up appointment, are aged 18 years or over, are able to read and write in English and to understand and sign the written information consent form. Patients will be ineligible if they are known to have a familial syndrome predisposing them to RCC, have had surgery for a second primary kidney cancer or have any other conditions which, in the opinion of the local Principal Investigator, makes them unsuitable for study participation.

## 5 METHODS

### 5.1 Participants and recruitment

We aim to recruit 60 participants from three centres across England and Scotland. Potentially eligible patients will be identified by members of the clinical team at each centre on a weekly basis by reviewing the appointment list for the clinic two to three weeks ahead or through the weekly multidisciplinary team meeting (MDT).

Potentially eligible patients will be sent a study pack including a cover letter on hospital‐headed paper, a participant information leaflet and a copy of the consent form. Contact details for the research team will be included in the participant information sheet and potential participants will be encouraged to contact the research team if they have any questions about the study. As participants may not know whether the excised renal mass is RCC in advance of the follow‐up consultation, the cover letter and participant information sheet will make clear that all those who have had surgery for a kidney mass are being invited and in the event that they do not in fact have cancer, they will be unable to participate in the study.

Patients potentially interested in taking part will be invited to arrive 20 minutes in advance of their follow‐up clinic appointment. Upon arrival and prior to their consultation, they will be met by a member of the clinical team who has been appropriately trained in obtaining consent for research studies. It will be explained to the patients that they can decide either to (i) participate in the study with their consultation being video and audio recorded; (ii) participate in the study with their consultation being just audio recorded; (iii) participate in the study without their consultation being video or audio recorded; or (iv) not participate in the study. For those agreeing to take part, written consent will be taken. The patients will then be given a corresponding card (green for (i), yellow for (ii), red for (iii) or no card at all for option (iv)) which they will present to their clinician upon walking into the consultation room.

**FIGURE 1:**
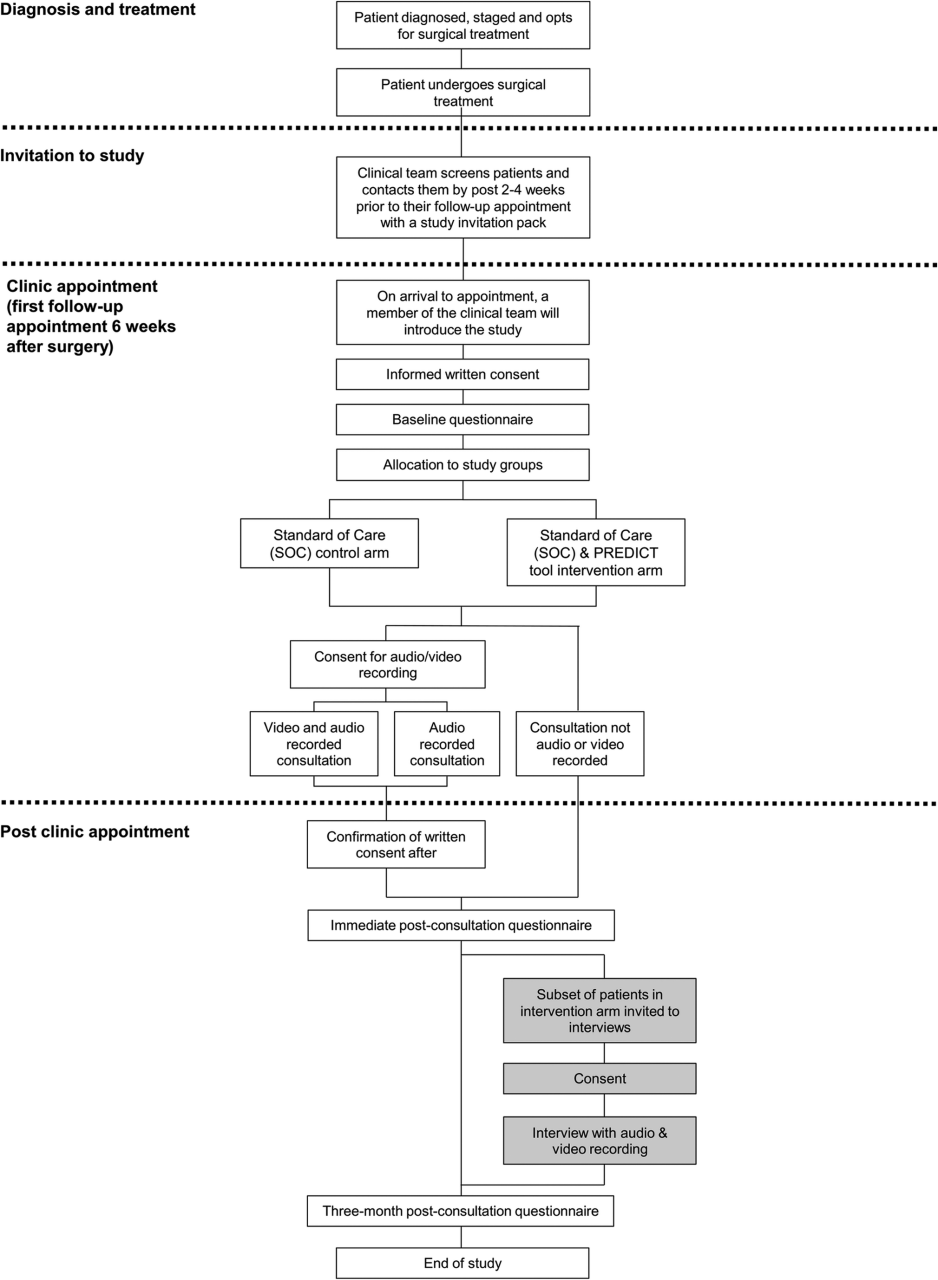
Patient pathway through study.

Participants will be then allocated to the ‘standard of care’ (SOC) arm or the ‘SOC and PREDICT Kidney’ (intervention) arm of the study (Figure 1). Allocation will be done such that patients at each site will be allocated to the SOC arm until a minimum of eight patients have been recruited. Subsequently, patients at each site will be allocated to the intervention arm. This approach has been chosen to reduce contamination between the arms by clinicians changing their SOC following participation in the intervention arm of the study.

Immediately following the consultation, participants in both arms of the study who consented to have their consultation audio and/or video recorded will have their consent for the use of that recording confirmed and will be asked to provide further written consent for the use of that data. If at that stage any participants do not wish us to keep the recordings, they will be deleted.

All clinicians based in the participating hospitals who currently conduct follow‐up consultations with patients following surgery for kidney cancer will be eligible to take part in the study. They will be invited to participate by the local Principal Investigators at each site and will be asked to consent to take part in the study. Additionally, they will be asked if they agree to have their consultations audio‐ or video‐recorded and if they consent to being contacted to arrange an interview.

### 5.2 Intervention

Patients in the SOC arm will have a consultation in which their pathology results, the risk of recurrence based on the Leibovich score and their follow‐up care plan are discussed. Within this usual care arm, patients are typically informed of the individual Leibovich score (0–11) and if this places them in the low, intermediate or high risk for recurrence group. They may also be told what the estimated average 5‐year absolute risk of recurrence for that risk group is as a percentage.

**FIGURE 2:**
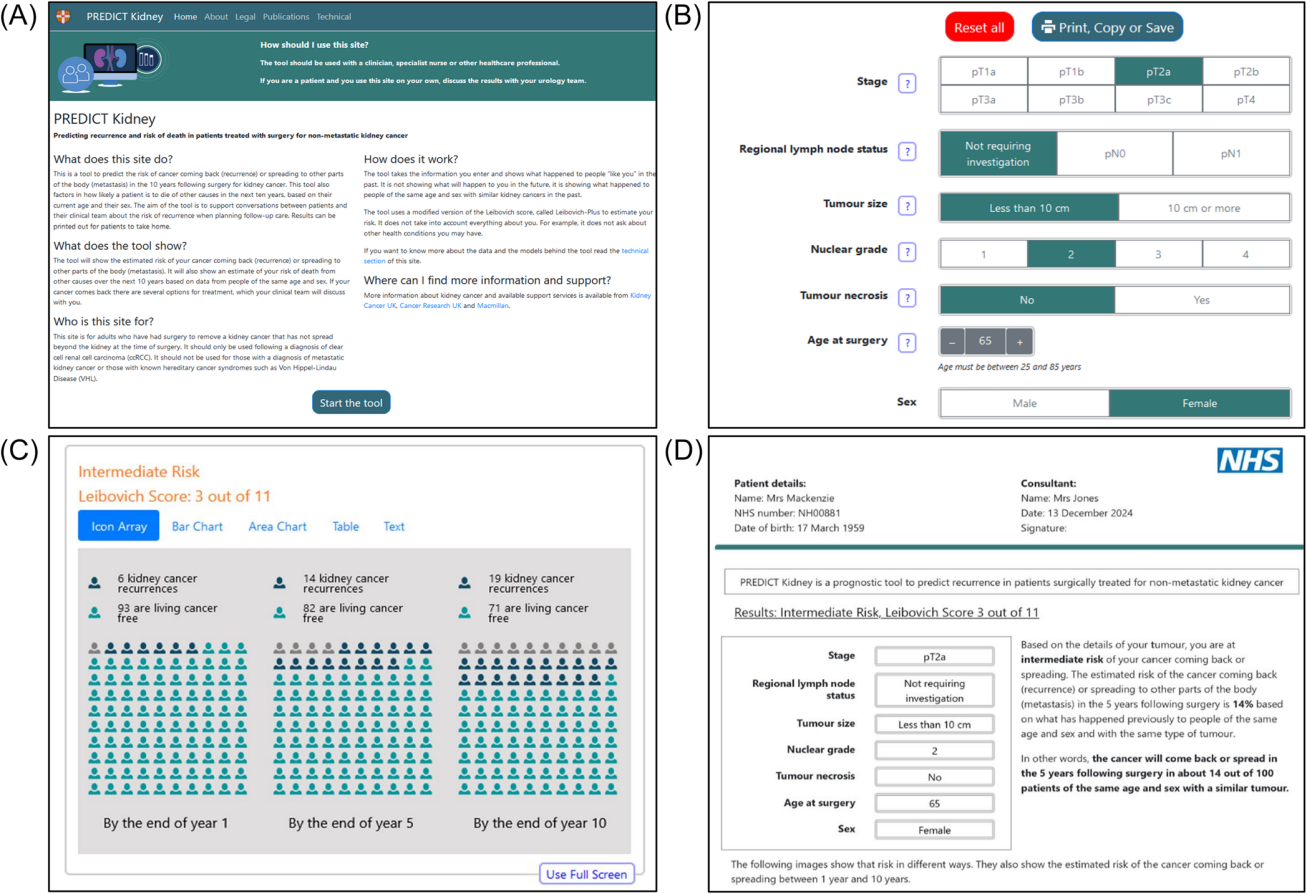
Screenshots of the PREDICT kidney tool: A) the landing page, B) the input section, C) icon array visualisation of kidney cancer recurrence, other cause of death and recurrence‐free survival, one of five visualisations available in the tool, and D) the information provided in the printout.

For patients in the intervention arm, the consultation will include the use of the new PREDICT Kidney tool to support the conversation about the risk of recurrence and risk‐stratified follow‐up. After the clinician has explained the pathology results and confirmed that the patient has kidney cancer, the clinician will enter the patient's risk factor information into the online PREDICT Kidney tool. The PREDICT Kidney tool is a risk‐communication tool that implements the augmented Leibovich model. It presents the risk of recurrence category based on the Leibovich score, alongside the 1‐year, 5‐year and 10‐year absolute risk of recurrence based on the Leibovich model and the 1‐year, 5‐year and 10‐year age‐ and sex‐matched absolute risk of death from other causes calculated using joint modelling with Cox proportional hazard models approach to include competing risks as applied in the development of the PREDICT Breast and PREDICT Prostate tools^18^, ^19^ (Figure 2).

Training on the PREDICT Kidney tool will be provided for the clinicians after the recruitment of control participants is complete to limit any contamination of the SOC consultations. The clinicians will be given a guidance document outlining how to best use the tool to communicate personalised risk estimates using the text and graphics generated. Each patient will be given a printed report from the tool at the end of their consultation including graphic results, what they mean, and links to more information and support. The design of the tool, the format of the risk visualisations and the report have been informed by the existing PREDICT Breast and PREDICT Prostate tools, the wider literature^20^ and a series of online workshops carried out by the research team. Workshops were conducted with: patients with experience of kidney cancer, members of the public without a history of kidney cancer and healthcare professionals involved in the care of patients with kidney cancer.

### 5.3 Sample size

We aim to recruit 30 patients to each arm (a total of 60). This will enable us to assess the recruitment rate to within +/− 10% with 95% confidence intervals (point estimate 50%). This number of participants is also sufficient to enable data collection for the qualitative components of the analysis.

### 5.4 Data collection

Before their consultation, study participants in both arms will be asked to complete a baseline paper questionnaire (File S1) asking about sociodemographic factors, health literacy,^21^ subjective and objective numeracy^22^, ^23^ and general health (measured using the EQ‐5D‐5L)^24^ (Table 2). Immediately following their consultation, study participants in both arms will be asked to complete a second paper questionnaire (File S2) including outcome measures relating to comprehension of risk of recurrence and follow‐up, perceived risk of cancer recurrence, risk conviction, satisfaction with the information provided and fear of cancer recurrence (Table 2). Subjective comprehension will be measured by averaging two Likert items asking: “How clear was the information you were given about ….? ” and “How much effort did you have to put in to understand the information you were given about ….? ”. Objective comprehension will be measured with a single question asking: “Which of these risk categories best describes your risk of cancer recurrence?”. Perceived risk of cancer recurrence will be measured using one question on a scale of 0–100 and a measure of risk conviction based on two questions used in previous studies.^25^ Fear of cancer recurrence will be measured using the first six questions from the Fear of Cancer Recurrence Inventory short form (FCRI‐SF)^26^).

**TABLE 2:** Details of measures and outcomes collected within patient questionnaires.

		Baseline	Immediate after consultation	3 months after consultation
**Sociodemographic**				
Age	10‐year age groups	•		
Sex	Female/Male/Prefer not to specify	•		
Gender	One question on whether the participant identifies with the same gender as sex registered at birth	•		
Ethnicity	Four categories plus Other/Prefer not to specify	•		
Postcode		•		
**Understanding of risk of recurrence**				
Subjective risk comprehension	Two questions on 7‐point scales		•	
Objective risk comprehension	Risk category and numeric 1–100 range		•	•
Risk conviction for numeric risk of recurrence	Two questions on 7‐point confidence scales^25^		•	•
Relative risk of recurrence and death from other causes	5‐point relative scale		•	•
Risk conviction for relative risk of recurrence and death from other causes	Two questions on 7‐point confidence scales^25^		•	•
**Understanding of follow‐up**				
Subjective Follow up Comprehension	Two questions on 7‐point scales		•	
**Fear of recurrence**				
Fear of recurrence	Fear of Cancer Recurrence Inventory short form (FCRI‐SF).^24^		•	•
**Satisfaction**				
Satisfaction with risk information	Two questions on 7‐point agreement scales		•	
Satisfaction with Follow up Information	Three questions on 7‐point agreement scales		•	
Overall Satisfaction with consultation	7‐point agreement scale		•	
**Views of the report** *				
Feeling	7‐point satisfaction scale		•	
Layout	7‐point satisfaction scale		•	
Information	7‐point satisfaction scale		•	
**Moderators**				
Subjective numeracy	Eight questions on 6‐point scale, taken from the cognitive ability domain of the Subjective Numeracy Scale^22^	•		
Objective numeracy	Three questions with numeric answers^23^	•		
Health Literacy	Single Item Literacy Screener (SILS)^21^	•		
General Health	EQ‐5D‐5L^24^	•		

* Intervention group only.

Three months after their consultation, study participants in both arms will be sent a final short questionnaire including measures of perceived risk of cancer recurrence and the complete Fear of Cancer Recurrence Inventory short form (FCRI‐SF) (Table 2, File S3).

To assess the fidelity of the use of the tool by clinicians and to monitor the additional time taken to deliver the intervention, a subset of both control and intervention consultations will be video recorded. A subset of study participants from the intervention arm will additionally be purposively sampled based on risk profile to take part in a telephone or online interview with a member of the research team within 2–6 weeks of their consultation. The interviews will be semi‐structured based on an interview schedule informed by the Theoretical Framework of Acceptability (TFA)^27^ and will include a discussion of the participants' experience of being told their risk of recurrence and competing risk of death from other causes using the PREDICT Kidney tool and how this has affected their understanding of their risk of recurrent disease.

All healthcare professionals participating in this study will also be invited to participate in an interview with a member of the research team. The interviews will be based on a schedule informed by the TFA,^27^ normalisation process theory^28^ and the system usability scale.^29^ They will focus on tool acceptability, perceived usability and perceived limitations and barriers to uptake.

### 5.5 Analysis

Descriptive statistics will be used to summarise recruitment into the study, characteristics of the study population at baseline, completeness of the questionnaires and quantitative measures within the questionnaires. Means and standard deviations will be used for normally distributed continuous variables, medians and interquartile range for non‐normally distributed continuous variables and numbers and percentages for categorical variables. Where reported, all proportions will be presented with 95% confidence intervals.

The impact of the PREDICT Kidney tool on patient‐level clinical outcomes will be tested first in an intention‐to‐treat analysis comparing the intervention group to the control group. A secondary *per protocol* analysis will then be performed. In both cases, for continuous outcome measures (satisfaction, fear of cancer recurrence and risk conviction), mean scores between groups at both time points will be compared using mixed linear regression models, adjusting for risk category and accounting for clustering within each site. All models will be adjusted for age, ethnicity, deprivation and EQ‐5D‐5L. The size of the difference between groups will be interpreted by comparison with the standard deviation (SD), with the criteria for clinically relevant change being a change of 0.5 SD. Exploratory subgroup analyses will be performed by the site to assess whether the location of the study had any impact on outcomes and to assess the variance of the outcomes, by the patient's risk category of recurrence and by age. Accuracy of perceived risk will be analysed as a binary outcome using logistic regression, again adjusted for risk category, clustering within each site and age, ethnicity, deprivation and EQ‐5D‐5L. Statistical significance will be p < 0.05. All analyses will be conducted in STATA 14 or R.

Audio from the video‐recorded consultations and interviews will be transcribed verbatim before analysis. Qualitative data from the semi‐structured interviews will be analysed using a mix of inductive and deductive Thematic Analysis. The inductive analysis will look for emergent themes relevant to the study aims and the deductive thematic analysis will be organised around the seven dimensions of the Theoretical Framework of Acceptability.^27^ Qualitative data from recorded consultations in the intervention arm will be used to assess clinician adherence to a fidelity checklist based on the ‘best practice’ guidance document provided to clinicians and the length of consultations.

## 6 DISCUSSION

Variations in current practice and the need for improved, consistent communication about the risk of recurrence of localised RCC following surgery have been identified by patients.^15^ The value of basing follow‐up care on individual‐level estimated risk of recurrence and considering competing risks has also been identified by researchers and clinicians.^11^ PREDICT Kidney is a new online tool developed to enable the estimation of these risks and to support clinicians to communicate the risk of recurrence so that patients feel more informed and have a better understanding of the rationale behind the planned follow‐up. While the use of risk prediction models to inform kidney cancer follow‐up regimes is recommended in current clinical guidance, to our knowledge, this is the first study to assess the use of a risk communication tool within routine care with this patient population.

A key strength of this study is the use of a tool that employs a well‐established risk model, the Leibovich model,^12^, ^30^, ^31^ employs visual aids to simplify communication of complex information in line with best practice,^32^ and has been developed with extensive input from those with experience of kidney cancer, members of the public and clinicians. By presenting an individual patient's estimated risk of death from other causes alongside their estimated risk of recurrence, our hypothesis is that the tool will facilitate more informed discussions around follow‐up care and encourage consideration of the whole person by the clinical team rather than the current RCC‐centric approach. While this study, given its size, will not enable us to demonstrate whether the use of the tool improves patient understanding of follow‐up or reduces fear of recurrence, the data collected will enable us to assess the feasibility and acceptability of incorporating the PREDICT Kidney online tool into the first post‐surgery follow‐up appointment for patients with localised RCC. In preparation for designing a future trial using this tool, we will evaluate participant recruitment, completeness of data collection and estimates of patient‐level outcomes.

Although the PREDICT Kidney tool currently employs an augmented version of the Leibovich model, if this study shows that the tool is acceptable to patients and clinicians and feasible to use within current clinical pathways, new or updated models with better performance in the target population,^33^ could be incorporated into the tool in the future. The tool could also be extended to support decisions around the newly available adjuvant treatment option for RCC patients. Alongside increasing evidence of the potential benefit of adjuvant treatment for those at intermediate or high risk of recurrence,^34^ there is an acknowledgement of the importance of informing patients about the potential benefits and harms of such treatment to facilitate shared decision‐making.^6^ By enabling communication of the absolute benefits and harms alongside the risk of death from other causes, the PREDICT Kidney tool could play an important role in supporting those conversations.

In conclusion, the PREDICT Kidney tool provides a significant opportunity to improve the follow‐up care of patients with RCC by offering a means to enhance risk communication and support personalised care strategies. The findings from this feasibility study will not only pave the way for a subsequent randomised controlled trial and further work to improve and extend the tool but will also offer valuable insights into the integration of digital tools into routine oncology practice. As the healthcare field continues to evolve towards personalised medicine, tools like PREDICT Kidney will be crucial in enabling the delivery of tailored and effective patient care.

## AUTHOR CONTRIBUTIONS

**Panayiotus Laouris:** Methodology; writing—original draft preparation. **Chiara Re:** Methodology; visualization; writing—review and editing. **Georgia Stimpson:** Methodology; software; visualization; writing—review and editing. **Axel Bex:** Conceptualisation; funding acquisition; writing—review and editing. **James Blackmur:** Methodology; writing—review and editing. **Alexander Laird:** Methodology; writing—review and editing. **Carley Batley:** Methodology; project administration; writing—review and editing. **Grant Stewart:** Conceptualisation; funding acquisition; methodology; visualization; writing—review and editing. **Hannah Harrison:** Conceptualisation; funding acquisition; methodology; software; supervision; visualization; writing—review and editing. **Juliet Usher‐Smith:** Conceptualisation; funding acquisition; methodology; supervision; visualization; writing—original draft preparation.

## CONFLICT OF INTEREST STATEMENT

GDS has received educational grants from Pfizer, AstraZeneca and Intuitive Surgical; consultancy fees from Pfizer, BMS, Merck, EUSA Pharma and CMR Surgical; travel expenses from BMS and Pfizer; and speaker fees from Pfizer. AB has received educational grants from Pfizer; consultancy fees from Pfizer, BMS, Roche and Telix; and speaker fees from Ipsen. AL has received consultancy fees from Intuitive Surgical and travel expenses and an educational grant from MSD.

## ETHICS STATEMENT

This study has received a favourable opinion from the East of England – Cambridge South Research Ethics Committee (REC reference 24/EE/0216).

## Supporting information


**File S1.** Baseline participant questionnaire.

**File S2.** Immediate follow‐up participant questionnaire (intervention group).

**File S3.** Three‐month follow‐up participant questionnaire.

## Data Availability

The pseudo‐anonymised data will be made available via the University of Cambridge data repository (https://www.repository.cam.ac.uk). Formal requests for access will be considered via a data‐sharing agreement that indicates the criteria for data access and conditions for research use and will incorporate privacy and confidentiality standards to ensure data security.
