# Supplementary material for: Feasibility study of using the PREDICT kidney tool for patients with localised renal cell carcinoma
Source: BJUI Compass. 2025 Mar 30;6(4):e70014. doi: 10.1002/bco2.70014 (PMC11955410; doi:10.1002/bco2.70014)
Supplement: Supplementary file 1 — File S1. Baseline participant questionnaire. [file BCO2-6-e70014-s002.pdf]

PREDICT Kidney Feasibility Trial
Baseline Questionnaire

This questionnaire is for participants of the ‘PREDICT Kidney Feasibility Trial’ and should be completed immediately before the follow-up consultation with your clinician.

Participation is voluntary and all information will remain confidential.

Please complete this questionnaire and then hand it to the member of the research team.

Thank you for your participation.

Your Details

Study ID ____

Date completing the questionnaire __________________

Study ID

Section 1: This first section covers some questions about your background and your general health to ensure we are capturing the views of people with a range of experiences and background.

Please check a **single** box corresponding to the answer that represents you the most.

1. How old are you?

☐ 18-20 ☐ 40-49 ☐ 70-79
☐ 20-29 ☐ 50-59 ☐ 80-89
☐ 30-39 ☐ 60-69 ☐ >90

2. What is your sex?

☐ Female
☐ Male
☐ Prefer not to specify

3. Is the gender you identify with the same as your sex registered at birth?

☐ Yes
☐ No – my gender identity is ___________________________
☐ Prefer not to specify

4. What is your ethnic group?

☐ White
☐ Mixed/Multiple ethnic group
☐ Asian/Asian British
☐ Black/African/Caribbean/Black British
☐ Other, please describe: __________________
☐ Prefer not to specify

5. What is the postcode of your current home address?

6. Under each heading, please tick the **ONE** box that best describes your health **TODAY**

MOBILITY

☐ I have no problems in walking about

☐ I have slight problems in walking about

☐ I have moderate problems in walking about

☐ I have severe problems in walking about

☐ I am unable to walk about

SELF-CARE

☐ I have no problems washing or dressing myself

☐ I have slight problems washing or dressing myself

☐ I have moderate problems washing or dressing myself

☐ I have severe problems washing or dressing myself

☐ I am unable to wash or dress myself

USUAL ACTIVITIES (e.g. work, study, housework, family or leisure activities)

☐ I have no problems doing my usual activities

☐ I have slight problems doing my usual activities

☐ I have moderate problems doing my usual activities

☐ I have severe problems doing my usual activities

☐ I am unable to do my usual activities

PAIN/DISCOMFORT

☐ I have no pain or discomfort

☐ I have slight pain or discomfort

☐ I have moderate pain or discomfort

☐ I have severe pain or discomfort

☐ I have extreme pain or discomfort

ANXIETY/DEPRESSION

☐ I am not anxious or depressed

☐ I am slightly anxious or depressed

☐ I am moderately anxious or depressed

☐ I am severely anxious or depressed

☐ I am extremely anxious or depressed

7. We would like to know how good or bad your health is TODAY. The following scale is numbered from 0 to 100. 100 means the <u>best</u> health you can imagine. 0 means the <u>worst</u> health you can imagine.

   a. Mark an X on the scale on the right to indicate how your health is TODAY.

   b. Now, please write the number you marked on the scale in the box below.

   YOUR HEALTH TODAY =

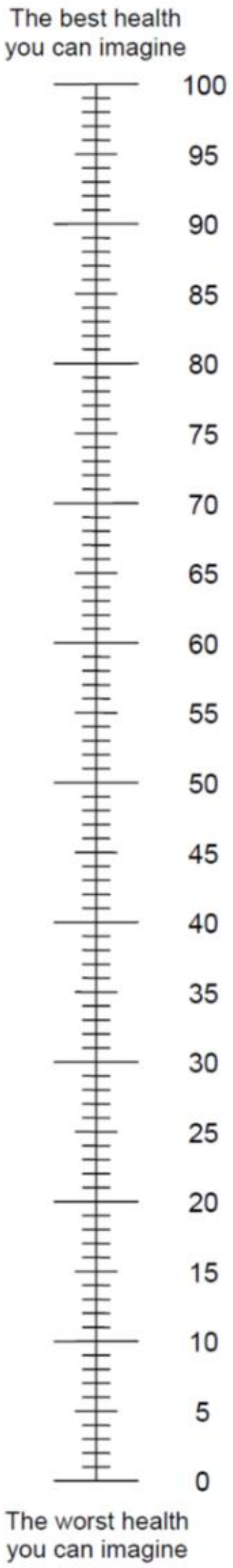

Section 2: This second section covers some questions about your preferences for numbers and information. It is not a test but is to help us understand how to present information in a way that makes most sense to different people.

8. How often do you need to have someone help you when you read instructions, leaflets, or other written material from your doctor or pharmacy?

☐ Never ☐ Sometimes ☐ Always

☐ Rarely ☐ Often

9. How good are you at working with fractions e.g. $\frac{1}{3}$?

Not at all good ☐ ☐ ☐ ☐ ☐ ☐ Extremely good

10. How good are you at working with percentages e.g. 10%?

Not at all good ☐ ☐ ☐ ☐ ☐ ☐ Extremely good

11. How good are you at calculating a 15% tip?

Not at all good ☐ ☐ ☐ ☐ ☐ ☐ Extremely good

12. How good are you at figuring out how much a shirt will cost if it is 25% off?

Not at all good ☐ ☐ ☐ ☐ ☐ ☐ Extremely good

13. When reading the newspaper, how **helpful** do you find tables and graphs as part of a story?

Not at all helpful ☐ ☐ ☐ ☐ ☐ ☐ Extremely helpful

14. When people tell you the chance of something happening, do you prefer that they use **words** ("it rarely happens") or **numbers** ("there's a 1% chance")?

Always Prefer Words ☐ ☐ ☐ ☐ ☐ ☐ Always Prefer Numbers

15. When you hear a weather forecast, do you prefer predictions using only **words** (e.g. "there is a small chance of rain today") or predictions using **percentages** (e.g. "there will be a 20% chance of rain today")?

Always Prefer Words ☐ ☐ ☐ ☐ ☐ ☐ Always Prefer Percentages

16. How **often** do you find numerical information to be useful?

Never ☐ ☐ ☐ ☐ ☐ ☐ Very often

17. Imagine we flip a fair coin 1000 times. What is your best guess at how many times the coin would come up heads in 1000 flips?

[ ]

18. In the UK National Lottery®, the chance of winning a £10 prize is 1%. What is your best guess at how many people would win a £10 prize if 1000 people each bought a single ticket to UK National Lottery®?

19. In the EuroMillions® Lottery, the chance of winning a car is 1 in 1000. What percent of the EuroMillions® tickets win a car?
