# Supplementary material for: Feasibility study of using the PREDICT kidney tool for patients with localised renal cell carcinoma
Source: BJUI Compass. 2025 Mar 30;6(4):e70014. doi: 10.1002/bco2.70014 (PMC11955410; doi:10.1002/bco2.70014)
Supplement: Supplementary file 2 — File S2. Immediate follow‐up participant questionnaire (intervention group). [file BCO2-6-e70014-s003.pdf]

PREDICT Kidney Feasibility Trial
Immediate Post-consultation Questionnaire

This questionnaire is for participants of the 'PREDICT Kidney Feasibility Trial' and should be completed at the end of the follow-up consultation with your clinician.

Participation is voluntary and all information will remain confidential.

When you finish, please hand the completed questionnaire over to the member of the research team. Thank you for your participation.

Participant Details (to be completed by Researcher)

Study ID ____________________________________

Questionnaire date______________________________

UNIVERSITY OF CAMBRIDGE

Cambridge University Hospitals NHS
NHS Foundation Trust

Section 1: In this first section we are interested in your thoughts on the information you received in the consultation.

Please read each statement below carefully, keeping in mind the medical care you received today, and **check** the single box that best represents whether you agree or disagree.

1. How much effort did you need to put in to understand the information you were given about the risk of the kidney cancer coming back (recurrence)?

   Not a lot ☐ ☐ ☐ ☐ ☐ ☐ ☐ A lot

2. How clear did you find the information you were given about the risk of the kidney cancer coming back?

   Not at all clear ☐ ☐ ☐ ☐ ☐ ☐ ☐ Completely clear

3. How satisfied are you with the amount of information you received about the risk of cancer recurrence?

   Not at all satisfied ☐ ☐ ☐ ☐ ☐ ☐ ☐ Extremely satisfied

4. How satisfied are you with the way in which you were given information about the risk of cancer recurrence?

   Not at all satisfied ☐ ☐ ☐ ☐ ☐ ☐ ☐ Extremely satisfied

5. How much effort did you need to put in to understand how your follow-up plan relates to your risk of the kidney cancer coming back?

   Not a lot ☐ ☐ ☐ ☐ ☐ ☐ ☐ A lot

6. How clear did you find the information you were given about how your follow-up plan relates to your risk of the kidney cancer coming back?

   Not at all clear ☐ ☐ ☐ ☐ ☐ ☐ ☐ Completely clear

7. How satisfied are you with the amount of information you received about your follow-up plan?

   Not at all satisfied ☐ ☐ ☐ ☐ ☐ ☐ ☐ Extremely satisfied

8. How satisfied are you with the way in which you were given information about the follow-up plan?

   Not at all satisfied ☐ ☐ ☐ ☐ ☐ ☐ ☐ Extremely satisfied

9. Do you feel you had enough information to take part in discussions about your follow-up plan?

   No, not at all ☐ ☐ ☐ ☐ ☐ ☐ ☐ Yes definitely

10. Overall, how satisfied were you with your follow-up consultation as a whole?

   Not at all satisfied ☐ ☐ ☐ ☐ ☐ ☐ ☐ Extremely satisfied

Section 2: In this section we are interested in your thoughts on the report you received during your consultation.

Please read each statement below carefully, keeping in mind the medical care you received today, and **check** the single box that best represents whether you agree or disagree.

	**Not at satisfied**						**Extremely satisfied**
How satisfied are you with....							
11. The general format (look and feel) of the report	☐	☐	☐	☐	☐	☐	☐
12. The amount of information on the report	☐	☐	☐	☐	☐	☐	☐
13. The way information is organised on the report	☐	☐	☐	☐	☐	☐	☐

Section 3: In this final section we are interested in how you feel about cancer recurrence.

Most people who have been diagnosed with cancer are worried, to varying degrees, that their cancer could return or progress in the same place or in another part of the body, something we refer to as recurrence. Please read each statement and indicate to what degree it applies to you by checking the most appropriate **single** answer for each statement.

	**Not at all**	**A little**	**Somewhat**	**A lot**	**A great deal**
14. I am worried or anxious about the possibility of cancer recurrence	☐	☐	☐	☐	☐
15. I am afraid of cancer recurrence	☐	☐	☐	☐	☐
16. I believe it is normal to be worried or anxious about the possibility of cancer recurrence	☐	☐	☐	☐	☐
17. When I think about the possibility of cancer recurrence, this triggers other unpleasant thoughts or images (such as death, suffering, the consequences for my family)	☐	☐	☐	☐	☐
18. I believe that I am cured, and that the cancer will not come back	☐	☐	☐	☐	☐

19. In your opinion, are you at risk of having a cancer recurrence?

☐ Not at all at risk

☐ A little at risk

☐ Somewhat at risk

☐ A lot at risk

☐ A great deal at risk

21. Which of these risk categories best describes your risk of cancer recurrence?

☐ Low risk

☐ Intermediate risk

☐ High risk

☐ I don't know

22. On a scale from 0 to 100% how likely do you think it is that your kidney cancer will come back within the next 5 years? Please write an estimated percentage in the box below.

| %
|---|

23. How certain are you about your answer to the above question?

Not at all certain	☐	☐	☐	☐	☐	☐	☐	Extremely certain

24. How confident are you that the estimate you have given of how likely do you think it is that your kidney cancer will come back within the next 5 years is accurate, that is, that it reflects your actual risk?

Not at all confident	☐	☐	☐	☐	☐	☐	☐	Extremely confident

25. How do you think the risk of your kidney cancer coming back within the next 5 years compares with your chance of dying from something else in that time?

☐ Much less

☐ Less

☐ About the same

☐ Greater

☐ Much greater

26. How certain are you about your answer to the above question?

Not at all certain	☐	☐	☐	☐	☐	☐	☐	Extremely certain

27. How confident are you that the estimate you have given of your kidney cancer coming back within the next 5 years compares with your chance of dying from something else in that time is accurate, that is, that it reflects your actual risk?

Not at all confident	☐	☐	☐	☐	☐	☐	☐	Extremely confident

28. Do you have any other comments or feedback?

____

____

____

____
