# Supplementary material for: Feasibility study of using the PREDICT kidney tool for patients with localised renal cell carcinoma
Source: BJUI Compass. 2025 Mar 30;6(4):e70014. doi: 10.1002/bco2.70014 (PMC11955410; doi:10.1002/bco2.70014)
Supplement: Supplementary file 3 — File S3. Three‐month follow‐up participant questionnaire. [file BCO2-6-e70014-s001.pdf]

PREDICT Kidney Feasibility Trial

Three-month post-consultation Questionnaire

Thank you very much for taking part in this study.

It is now three months since your first follow-up consultation after surgery for your kidney cancer. To help us understand how you are feeling now, we would be really grateful if you could complete this short two-page questionnaire and return it to us in the pre-paid envelope. If you have any questions or concerns or need help completing the questionnaire, please contact ###.

Your Details

Study ID ____________________

Date completing the questionnaire ____________

UNIVERSITY OF CAMBRIDGE

Cambridge University Hospitals NHS
NHS Foundation Trust

**Study ID**	

In this questionnaire we are interested in how you feel about cancer recurrence.

Most people who have been diagnosed with cancer are worried, to varying degrees, that their cancer could return or progress in the same place or in another part of the body, something we refer to as recurrence. Please read each statement and indicate to what degree it applied to you **during the past month** by checking the most appropriate **single** answer for each statement.

	Not at all	A little	Somewhat	A lot	A great deal
1. I am worried or anxious about the possibility of cancer recurrence	☐	☐	☐	☐	☐
2. I am afraid of cancer recurrence	☐	☐	☐	☐	☐
3. I believe it is normal to be worried or anxious about the possibility of cancer recurrence	☐	☐	☐	☐	☐
4. When I think about the possibility of cancer recurrence, this triggers other unpleasant thoughts or images (such as death, suffering, the consequences for my family)	☐	☐	☐	☐	☐
5. I believe that I am cured, and that the cancer will not come back	☐	☐	☐	☐	☐

6. In your opinion, are you at risk of having a cancer recurrence?

☐ Not at all at risk
☐ A little at risk
☐ Somewhat at risk
☐ A lot at risk
☐ A great deal at risk

7. How often do you think about the possibility of cancer recurrence?

☐ Never
☐ A few times a month
☐ A few times a week
☐ A few times a day
☐ Several times a day

8. How much time **per day** do you spend thinking about the possibility of cancer recurrence?

☐ I don’t think about it
☐ A few seconds
☐ A few minutes
☐ A few hours
☐ Several hours

10. How long have you been thinking about the possibility of cancer recurrence?

☐ I don't think about it

☐ A few weeks

☐ A few months

11. Which of these risk categories best describes your risk of cancer recurrence?

☐ Low risk

☐ Intermediate risk

☐ High risk

☐ I don't know

12. On a scale from 0 to 100% how likely do you think it is that your kidney cancer will come back within the next 5 years? Please write an estimated percentage in the box below.

[%]

13. How certain are you about your answer to the above question?

Not at all certain ☐ ☐ ☐ ☐ ☐ ☐ ☐ Extremely certain

14. How confident are you that the estimate you have given is accurate, that is, that it reflects your actual risk?

Not at all confident ☐ ☐ ☐ ☐ ☐ ☐ ☐ Extremely confident

15. How do you think the risk of your kidney cancer coming back within the next 5 years compares with your chance of dying from something else in that time?

☐ Much less

☐ Less

☐ About the same

☐ Greater

☐ Much greater

16. How certain are you about your answer to the above question?

Not at all certain ☐ ☐ ☐ ☐ ☐ ☐ ☐ Extremely certain

17. How confident are you that the estimate you have given is accurate, that is, that it reflects your actual risk?

Not at all confident ☐ ☐ ☐ ☐ ☐ ☐ ☐ Extremely confident
